# Sintilimab for the treatment of non-small cell lung cancer

**DOI:** 10.1186/s40364-022-00363-7

**Published:** 2022-04-18

**Authors:** Lin Zhang, Weihao Lin, Fengwei Tan, Ning Li, Qi Xue, Shugeng Gao, Yibo Gao, Jie He

**Affiliations:** 1 Department of Thoracic Surgery, National Cancer Center/National Clinical Research Center for Cancer/Cancer Hospital, Chinese Academy of Medical Sciences and Peking Union Medical College, Beijing, China; 2 Department of Oncology, Renmin Hospital of Wuhan University, Wuhan, China; 3 Department of Good Clinical Practice Center, National Cancer Center/National Clinical Research Center for Cancer/Cancer Hospital, Chinese Academy of Medical Sciences and Peking Union Medical College, Beijing, China; 4 State Key Laboratory of Molecular Oncology, National Cancer Center/National Clinical Research Center for Cancer/Cancer Hospital, Chinese Academy of Medical Sciences and Peking Union Medical College, Beijing, China; 5 Laboratory of Translational Medicine, National Cancer Center/National Clinical Research Center for Cancer/Cancer Hospital, Chinese Academy of Medical Sciences and Peking Union Medical College, Beijing, China; 6 Central Laboratory, National Cancer Center/National Clinical Research Center for Cancer/Cancer Hospital & Shenzhen Hospital, Chinese Academy of Medical Sciences and Peking Union Medical College, Shenzhen, China

**Keywords:** PD-1, PD-L1, Sintilimab, Immunotherapy, Biomarker, Non-small cell lung cancer

## Abstract

Anti-programmed death-1 (PD-1)/programmed death-ligand 1 (PD-L1) immunotherapy has dramatically changed the therapeutic landscape of inoperable non-small cell lung cancer (NSCLC), and has been included in first-line treatments. Sintilimab is a domestic anti-PD-1 monoclonal antibody in China that has received approvals from the National Medical Products Administration to treat classical Hodgkin’s lymphoma, hepatocellular carcinoma, and squamous and non-squamous NSCLC. In a prospective clinical study we led, neoadjuvant sintilimab has led to major and complete pathologic responses, which are recommended as surrogate endpoints for neoadjuvant immunotherapy; however, its effect remains inconclusive in pulmonary ground glass nodules. Meanwhile, combination plans seem more likely to be satisfying therapeutic options. Specifically, sintilimab plus platinum-based chemotherapy plans conferred better anti-tumor efficacy and clinical benefits compared to chemotherapy alone, which led to their approval in China and the acceptance of a biological license application in the US. Besides, the combination with other plans, such as docetaxel, cytokine-induced killer cell immunotherapy, radiation therapy, and anlotinib have also shown promising anti-tumor efficacy, with acceptable toxicities, and are therefore worth further exploration. In addition, several clinical trials on NSCLC at our center are ongoing. In general, sintilimab and its combinatorial plans were effective and well tolerated, but the treatment requires appropriate timing; pathologic responses can be surrogate endpoints for neoadjuvant immunotherapy, while more effective biomarkers are warranted. This study provides an overview of sintilimab-based clinical trials on NSCLC, and may support further investigation of sintilimab in future clinical trials.

## Introduction

Lung cancer is the second most common in terms of new cancer cases and is the leading cause of cancer-related fatality globally [1]. Non-small cell lung cancer (NSCLC) comprises more than 85% of diagnosed lung cancer cases, and approximately 70% of the diagnosed NSCLC patients are the non-squamous type [2]. Surgical excision remains the optimal treatment option for NSCLC when the tumor is resectable; meanwhile, preoperative and postoperative chemotherapy significantly improves patient prognosis. On disease progression, platinum-based doublet chemotherapy used to be a first-line treatment for patients without a targetable driver oncogene [3–6].

During the last decade, the emergence of immune checkpoint inhibitors (ICI) has radically changed the landscape of NSCLC treatment. Immune checkpoints are co-stimulating surface proteins on T cells that transmit inhibitory signals [7]. Through interacting with immune checkpoints, tumor cells can retard the activation of T cells and their cytotoxic effects on tumors, leading to immune evasion [7]. As cell surface receptor proteins, immune checkpoints can be easily inhibited by antibodies. Among these antibodies, anti-programmed death-1 (PD-1)/programmed death-ligand 1 (PD-L1) antibodies are the most successful and have been approved to treat a wide variety of cancers, such as blood, skin, lung, liver, bladder and kidney cancers [8, 9].

Sintilimab is a domestic fully human IgG4 monoclonal antibody against PD-1 used in China [10, 11]. It was first approved by the National Medical Products Administration (NMPA) for the treatment of classical Hodgkin’s lymphoma patients who have relapsed or are refractory after ≥ 2 lines of systemic chemotherapy [10, 11]. Afterwards, sintilimab received approvals from the NMPA to be used in NSCLC in combination with chemotherapy as first-line therapies, and in hepatocellular carcinoma with IBI305 as a first-line therapy [12–14]. Previously, we led a phase Ib study (ChiCTR-OIC-17013726) on NSCLC to evaluate the effect of sintilimab on resectable NSCLC in a neoadjuvant setting, and the 2-year follow-up result has been updated in the American Society of Clinical Oncology meeting library [15, 16]. Furthermore, results of important phase 3 studies on NSCLC (ORIENT-11 and ORIENT-12) have been released recently and provided bases for new approvals for regimens containing sintilimab as first-line therapies [17, 18]. In addition, three clinical trials at our center are ongoing. The drug has shown promising prospects in treating NSCLC. Therefore, we comprehensively reviewed the prospective clinical trials to illustrate the efficacy, safety and potential predictive biomarkers of sintilimab-containing plans in NSCLC.

## Overview of PD-1/PD-L1 approvals in NSCLC

Checkpoint inhibitors have been approved and recommended in first-line therapies in advanced or metastatic NSCLC [19]. PD-L1 expression is currently the best, yet not optimal, biomarker for assessing whether patients are candidates for PD-1/PD-L1 inhibitors [20]. For patients who lack a driver mutation and have a tumor PD-L1 expression ≥50%, the National Comprehensive Cancer Network (NCCN) recommended single-agent pembrolizumab for all NSCLC, or pembrolizumab plus pemetrexed and platinum (PP) for non-squamous NSCLC, pembrolizumab plus gemcitabine and platinum (GP) for squamous NSCLC (preferred plan); for patients with a PD-L1 expression of 1–49%, pembrolizumab plus PP for non-squamous NSCLC, and pembrolizumab plus GP for squamous NSCLC [20, 21]. Meanwhile, in patients with PD-L1 ≥ 1%, nivolumab, atezolizumab and cemiplimab are also included in first-line therapeutic plans [19].

In the USA, there are six PD-1/PD-L1 inhibitors approved by the FDA to treat NSCLC; among them, pembrolizumab, nivolumab and cemiplimab are PD-1 blockers; and durvalumab, avelumab and atezolizumab are PD-L1 blockers [22]. In China, nivolumab, pembrolizumab, durvalumab, atezolizumab, camrelizumab, tislelizumab and sintilimab have been approved by the NMPA to treat NSCLC. The approved PD-1/PD-L1 inhibitors in NSCLC have been discussed elsewhere and well summarized by Gan et al. and Sanaei et al. [22, 23].

## Pharmacodynamics and pharmacokinetics

Sintilimab is an IgG4 anti-PD-1 monoclonal antibody generated using yeast display technology and developed for cancer treatment [24]. It has an IgG4 backbone, an antibody isotype that is known to have very low effector function and is an ideal choice for therapeutic antibodies. Structural analysis showed that the epitope of the sintilimab/PD-1 complex is located at the FG loop of PD-1, which is different from that of nivolumab or pembrolizumab [25]. Sintilimab has a higher binding affinity compared to nivolumab or pembrolizumab, with the dissociation constants being 74 pM, 3186 pM and 1785 pM, respectively [26]. In vitro, compared to nivolumab or pembrolizumab, sintilimab was able to bind with more PD-1 molecules on CD3 + T cells, and has better T cell activating characteristics [26]. In accordance with this, a sustained high PD-1 receptor occupancy (> 95%) was observed in patients 4 weeks after a single intravenous infusion of sintilimab [26].

Pharmacokinetic and anti-drug antibody (ADA) analyses of sintilimab have been undertaken in cells, animals and humans. In vitro, sintilimab exhibited no antibody-dependent cell-mediated cytotoxicity and complement-dependent cytotoxicity function [24]. In cynomolgus monkeys, the serum concentration of sintilimab and the area under the curve increased in a dose-dependent manner between 1 and 30 mg/kg, while ADAs were detected in nearly all of the animals. Sintilimab was well tolerated when administrated biweekly up to 200 mg/kg and caused no drug-related deaths [24]. In human PD-1 knock-in mice, subsequent to a single intravenous administration at 10 mg/kg, standard pharmacokinetic measurements of sintilimab, nivolumab and pembrolizumab serum concentrations indicated a serum half-life of 35.6, 43.5 and 42.5 h, respectively [26]. In 381 patients treated with sintilimab, only 0.52% (2/381) were detected as ADA-positive, and 0.26% (1/381 patients) developed neutralizing antibodies after sintilimab infusion [26].

The potential anti-tumor effect of sintilimab was also investigated in vitro and in vivo. When incubated with monocyte-derived dendritic cells and allogeneic whole CD4 + T cells, sintilimab significantly increased the levels of IL-2 and IFN-γ in a concentration-dependent manner. However, sintilimab does not have a significant direct agonistic effect on immune cells; in whole blood treated with sintilimab, cytokine profiles were not significantly changed [24]. In vivo, the anti-tumor efficacy of sintilimab was confirmed in a therapeutic human PD-1 knock-in tumor mouse model of MC38 colon adenocarcinoma [24]. In a human tumor xenograft model in NOG mice reconstituted with human immune cells, sintilimab showed an effective tumor inhibition effect at 1 mg/kg and 10 mg/kg compared to nivolumab or pembrolizumab [26]. Due to the preliminarily confirmed efficacy and safety profile in preclinical studies, sintilimab was tested in a series of clinical trials.

## Clinical trials of sintilimab

**Fig. 1:**
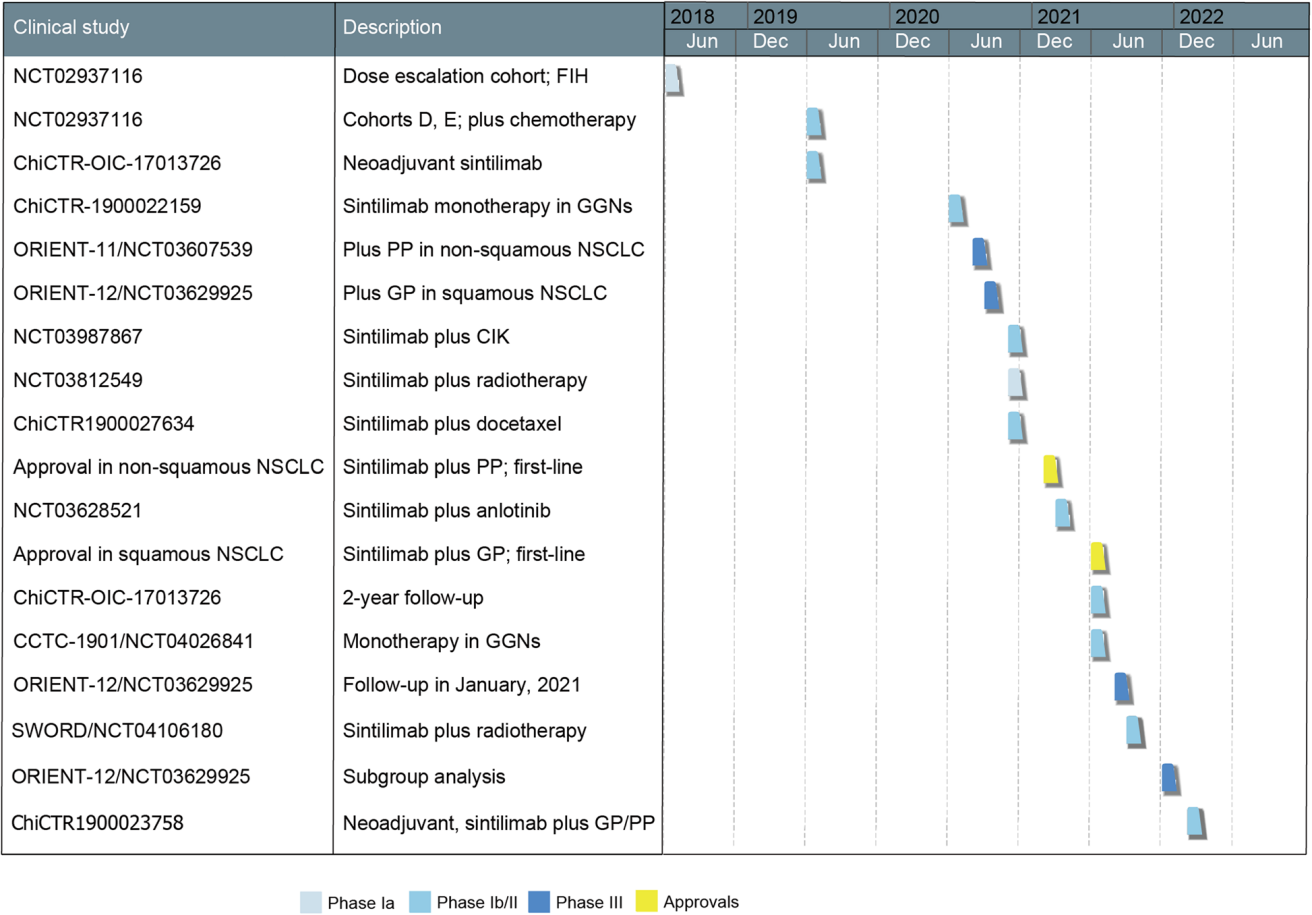
Timeline of clinical trials and approvals of sintilimab on non-small cell lung cancer. Dates indicate the time when the data of the research were first available online. FIH, first-in-human study; GGN, ground-glass nodule; PP, platinum plus pemetrexed; GP, platinum plus gemcitabine; NSCLC, non-small cell lung cancer; CIK, cytokine-induced killer cell immunotherapy

**Fig. 2:**
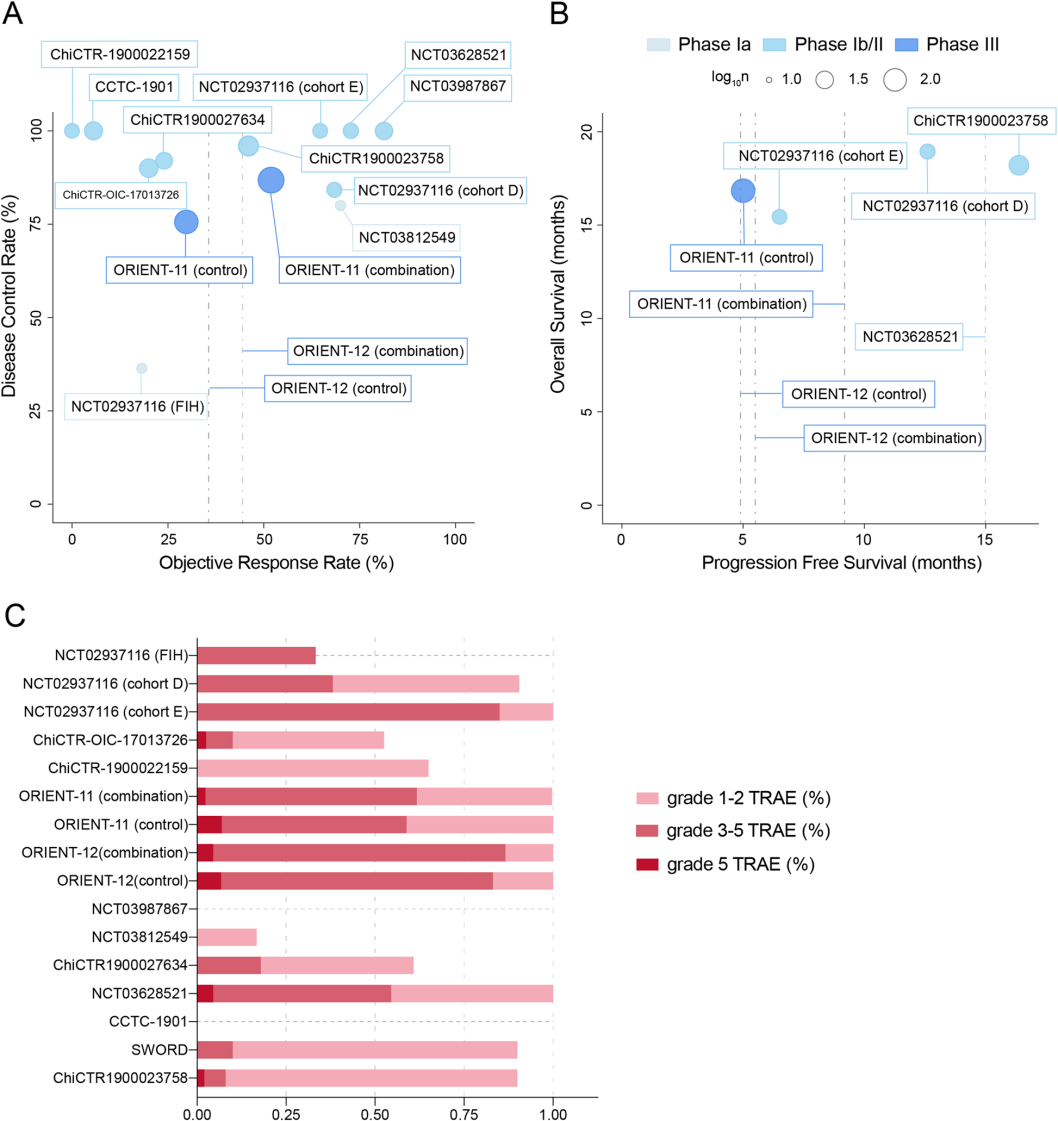
Anti-tumor effects (**A**, **B**) and incidence of adverse effects (**C**) from clinical trials of sintilimab on non-small cell lung cancer patients. Dashed lines indicate absence of available corresponding data

Sintilimab was first tested in patients with Hodgkin’s lymphoma in March 2017 [27]. In December 2017, the first study that investigated the effects of PD-1 blockade in a neoadjuvant setting in NSCLC in the Chinese population was carried out at our center [15, 16]. This is also the first clinical study of sintilimab in lung cancer, results of further clinical studies have been reported since then. Information of the studies is shown in Fig. 1, Fig. 2 and discussed below.

### Monotherapies

#### First-in-human study

﻿The preliminary safety and optimal dosage of sintilimab have been confirmed in the first-in-human study NCT02937116 in 2018. In NCT02937116, 12 patients with advanced solid tumors were included and given single-agent sintilimab at escalating dose levels [28]. During the treatment, four patients experienced grade 3 or higher adverse events (AE) and no treatment-related deaths were observed [28]. The recommended dosage was confirmed as 200 mg Q3W and a maximum dosage of 10 mg/kg could be well-tolerated; there were two patients with partial response (PR), and two with stable disease (SD) [28]. With an acceptable safety profile, a confirmed dosage regimen and a preliminary anti-tumor effect, sintilimab was further studied in later phase clinical trials.

#### Sintilimab as a neoadjuvant therapy

Neoadjuvant therapy has shown clinical benefit in resectable NSCLC patients and is recommended by the NCCN guidelines [19]. In 2014, a published meta-analysis that included 15 randomized controlled trials and 2385 operable patients showed that significant benefits were achieved with preoperative chemotherapy in terms of overall survival and disease-free survival [4]. Research exploring sintilimab as a neoadjuvant therapy has also been conducted. We led the single-center, single-arm, phase Ib study ChiCTR-OIC-17013726, in which sintilimab was tested as a neoadjuvant therapy in 40 treatment-naive patients with stage IA–IIIB NSCLC [15]. All patients received two cycles of sintilimab and 37 received surgery, among whom 36 then underwent R0 resection. Major pathological response (MPR) was achieved in 15 patients (40.5%) and pathologic complete response (pCR) was achieved in six patients (16.2%) [15]. Among the 40 patients, eight achieved a radiological PR, 28 achieved SD and four had progressive disease (PD) [15]. The PD-L1 expression in stromal cells at the primary site at baseline was correlated with the percentage of pathologic response of the primary lesion [15]. At the two-year follow-up, a total of 12 (33.3%) patients experienced relapse, and six patients had died; the median follow-up for disease-free survival (DFS) and overall survival (OS) for all 40 enrolled patients was 23.9 months and 26.4 months; the median DFS and OS were both not reached for the R0 resection group [16]. Interestingly, in the initial assessment, all patients with MPR or pCR were diagnosed with squamous cell NSCLC; consistent with this, the DFS of patients with non-squamous NSCLC tended to be shorter than that of patients with squamous NSCLC, with a hazard ratio (HR) of 2.71 (*P* = 0.1479) [15, 16]. Thus, sintilimab may be a promising neoadjuvant therapeutic strategy for resectable NSCLC and result in a favorable pathologic response, while patients with a squamous pathologic type might benefit more from it.

#### Sintilimab in multiple ground-glass nodules

A ground-glass nodule (GGN) is defined as an opacity of the lung on chest CT, without obscuring the bronchial and vascular margins [29]. In cases of GGNs, there is a major concern that they may be a sign of a primary lung cancer, with a reported malignancy rate ranging from 18 to 63% [30, 31]. Surgical resection is recommended for early-stage lung cancer patients, but the effect of PD-1 antibodies in early-stage lung cancer remains to be of great interest, yet inconclusive [32]. Thus, clinical studies have been performed in order to answer this question. In an exploratory phase I study ChiCTR-1900022159, 20 patients with at least one high-risk residual GGN after surgical resection of stage I NSCLC were included. All patients experienced stable disease after receiving sintilimab for 10 cycles, 6 weeks after surgery. Six patients were re-operated safely and there were 12 resected GGNs, in which pathology showed that one had no residual tumor cells, four had 20–35%, and seven had over 80% [33]. Interestingly, the highest tumor mutational burden (TMB, 15.7 mutations/Mb) was observed in the GGN with a complete response [33]. In addition, a single-center, phase II clinical trial (CCTC-1901/NCT04026841) was performed to confirm the effect of sintilimab on patients with multiple GGNs, and ORR was the primary endpoint [34]. Among the 36 patients included, all resected lesions were adenocarcinomas; CR was achieved in one patient, PR was achieved in one patient, and no grade 3–5 TRAEs were observed [34]. The proportion of CD8 + T cells and the ratio of CD8 + /CD4 + cells in unresected lesions of PR patients were significantly higher compared to those with SD lesions at baseline [34]. The proportions of patients achieving disease control were high in both studies. However, considering the indolent nature of pulmonary GGNs, the effect of sintilimab remains inconclusive.

### Combination therapies

#### Sintilimab in combination with platinum doublet

It has been demonstrated that addition of PD-1/PD-L1 antibodies to chemotherapy as a first-line therapy could offer extra survival benefits in NSCLC patients [35]. As a consequence, in the latest NCCN guidelines, platinum-based regimens in combination with a PD-1 inhibitor (pembrolizumab) became the preferred standard first-line treatment for unresectable or metastatic NSCLC [19]. Similar combinations were also investigated using sintilimab. Cohorts D and E of the multi-center, open-label, phase Ib trial NCT02937116 explored the efficacy of sintilimab plus PP in non-squamous NSCLC, and sintilimab plus GP in squamous NSCLC [36]. The primary endpoints were objective response rate (ORR), defined as the proportion of patients who achieved a complete remission (CR) or PR, and safety profile. The ORR of cohort D was 68.4% (13/19) and that of cohort E was 64.7% (11/17) [36]. Twenty-five patients developed grade 3 or higher TRAEs, and no treatment-related deaths were reported in either cohort [36]. In both groups, neither the expression of PD-L1 nor TMB was significantly correlated with treatment response. However, the TCR change clonality index was significantly higher in DCR patients compared to PD patients, the TCR change diversity index was significantly correlated with OS, and the TCR change clonality index was significantly correlated with PFS [36]. Based on this phase Ib trial, the two combination plans were further studied separately in two phase III clinical trials.

For non-squamous NSCLC, the anti-tumor effects of sintilimab plus PP were evaluated in the ORIENT-11/NCT03607539 trial [17, 37]. In the randomized, double-blind, phase III study ORIENT-11, 397 histologically or cytologically confirmed stage IIIB to IV non-squamous NSCLC patients were randomly assigned (2:1) to receive either sintilimab plus PP (sintilimab group), or placebo plus PP (placebo group) [17]. The primary endpoint was PFS, and the median PFS was significantly longer in the sintilimab group compared to the placebo group (8.9 months versus 5.0 months, HR = 0.482, *P* < 0.00001), while median OS was not reached [17]. Despite the fact that the sintilimab plus PP plan was correlated with better efficacy, it caused less fatality. AEs that led to death occurred in six patients (2.3%) in the sintilimab-combination group and in nine patients (6.9%) in the placebo-combination group [17]. In patients with PD-L1 < 1%, the median PFS was not significantly different between the two groups, while in patients with PD-L1 > 1%, the differences were significant; ORRs were significantly different between the sintilimab group and placebo group in all PD-L1 subgroups[17]. In the updated follow-up of ORIENT-11, researchers found that median OS was significantly improved in the sintilimab group compared with the placebo group (not reached versus 16.8 months, *p* = 0.0003) [37]. Furthermore, the patient-reported outcomes of ORIENT-11 showed that the addition of sintilimab to standard platinum-based chemotherapy delayed worsening of lung cancer symptoms, alleviated pain symptoms, and maintained life quality of these patients. The updated biomarker analyses of ORIENT-11 revealed that, while a higher PD-L1 expression [cutoff value of tumor proportion score (TPS) 1 and TPS50] was related to better PFS, the MHC class II antigen presentation pathway and CIITA were strongly associated with improved PFS and OS despite PD-L1 expressions. The immune infiltration analyses revealed that high or medium immune cell infiltration was strongly associated with improved PFS in the sintilimab group [37]. In summary, the results showed that the addition of sintilimab to chemotherapy resulted in significantly longer PFS and OS in non-squamous NSCLC, which led to its approval in non-squamous NSCLC as a first-line therapy in February, 2021 [14]. In addition, the clinical benefit conferred by this combination plan resulted in the acceptance of a biological license application as a first-line therapy by the FDA in May, 2021 [38].

For squamous NSCLC, the anti-tumor effect of sintilimab plus GP was evaluated in ORIENT-12/NCT03629925 and that of sintilimab plus paclitaxel/nab-paclitaxel and platinum (TP) was also evaluated [18, 39]. In the randomized, double-blind, phase III ORIENT-12 study, 357 histologically or cytologically confirmed clinical stage IIIB to IV squamous NSCLC patients without EGFR-sensitive mutations or ALK rearrangements were enrolled and randomly assigned (1:1) to be administered either sintilimab plus GP (sintilimab group) or placebo plus GP (placebo group) [18]. The primary endpoint was PFS; median PFS of 5.5 months in the sintilimab group and 4.9 months in the placebo group (*P* < 0.00001) was observed at the data cutoff on March 25, 2020 [18]. As one of the secondary endpoints, OS was not reached in both groups, but the sintilimab group exhibited a better trend in outcome compared to the placebo group. The interim analysis also revealed a higher 6-month OS rate in the sintilimab group compared to the placebo group (91.8% versus 80.6%) [18]. Counterintuitively, TRAE-related deaths were less frequent in the sintilimab group (4.5%, 8/179) compared to the placebo group (6.7%, 12/178). Moreover, in subgroup analyses, sintilimab group showed benefit over placebo group for PFS and ORR in both disease staging subgroups (III or IV), irrespective of the PD-L1 expression level, and in patients with brain metastasis; the benefit was also observed for PFS in patients without brain metastasis and those with/without liver metastasis [40]. Thus, a clinical benefit was confirmed in the addition of sintilimab to standard GP chemotherapy compared with GP treatment alone. The combination plan was also approved in squamous NSCLC by NMPA in June, 2021. In a retrospective real-world data study conducted at the First Affiliated Hospital, Guangzhou Medical University, enrolled squamous NSCLC patients were treated with sintilimab plus TP (arm A) or sintilimab plus GP (arm B); the confirmed ORR was 59.4% (19/32) in arm A and 40.0% (8/20) in arm B, and the median OS was 21.3 months and 13.3 months [39].

The combination of sintilimab and chemotherapy has also been tested in a neoadjuvant setting. In the phase 2 study ChiCTR1900023758, NSCLC patients were given sintilimab plus GP or sintilimab plus PP as a neoadjuvant therapy for 2 to 4 cycles [41]. Among the enrolled patients, 46.0% (23/50) achieved PR and 50.0% (25/50) achieved SD; among 30 patients receiving surgery, 43.3% (13/30) achieved MPR and 20.0% (6/30) achieved pCR [41]. During the treatment, 1 patient died of grade 5 hepatic failure [41].

#### Sintilimab in combination with docetaxel

Docetaxel is one of the first-line chemotherapy agents used for NSCLC [19]. In a single-arm phase II study, ChiCTR1900027634, advanced NSCLC patients who had failed to respond to standard platinum doublet therapy and had not received ICIs previously were enrolled and given docetaxel plus sintilimab every 3 weeks for 4–6 cycles, followed by sintilimab maintenance until disease progression, unacceptable toxicity, or up to 2 years [42]. The estimated median PFS, the primary endpoint, was 5.5 months; the overall response rate, one of the secondary endpoints, was 24% (6/25) [42]. Encouragingly, there were no AEs leading to discontinuation or death. Therefore, the preliminary analysis of the study has shown encouraging efficacy and a tolerable safety profile of sintilimab plus docetaxel in NSCLC patients. Biomarker research of this study revealed that circulating tumor DNA (ctDNA) residual at sixth week (2 courses of treatment) was an independent risk factor for progression or death (HR = 100), and patients with positive ctDNA at 6th week had inferior OS and PFS [43]. In addition, the combination of sintilimab and docetaxel is being evaluated in the treatment of advanced or metastatic NSCLC patients who have failed to respond to previous platinum-containing chemotherapy in a center in China (NCT03798743, NCT04144582) [44]. In addition, sintilimab or placebo in combination with docetaxel or pemetrexed is being evaluated at another center (NCT03863483, NCT03830411) [44]. These studies may provide support for future approval of this plan.

#### Sintilimab in combination with cytokine-induced killer cell immunotherapy and chemotherapy

Cytokine-induced killer (CIK) cells are a heterogeneous population of T lymphocytes that are generated in vitro from peripheral blood mononuclear cells cultured with specific cytokines [45]. CIK cells have been recognized as a new type of anti-tumor effector cells and CIK cell immunotherapies have exhibited anti-tumor effects in various tumors [46]. The combination of CIK cell immunotherapy and chemotherapy has been shown to improve OS and PFS compared to chemotherapy alone in NSCLC patients [47].

Recently, a single-arm phase Ib study (NCT03987867) investigating autologous CIK cell immunotherapy in combination with sintilimab plus chemotherapy in patients with advanced NSCLC was conducted [48]. In this trial, 16 treatment-naive patients with stage IIIB–IV NSCLC received sintilimab plus platinum-based chemotherapy, and intravenous autologous CIK cells every 3 weeks until PD or intolerable toxicity [48]. Among 32 evaluable patients, the ORR and DCR were 81.3% and 100%, respectively. Despite the encouraging efficacy, the combination plan resulted in at least one fatal pneumonia among 34 treated patients. Nevertheless, the combination plan warrants further studies and the NCT03987867 study is still ongoing. The plan is now undergoing further investigation as a first-line treatment in stage IV NSCLC patients (NCT04836728) [44]. These results may give hope to late-stage NSCLC patients.

#### Sintilimab in combination with radiation therapy

Radiation therapy has played a predominant role in treating early and locally advanced NSCLC [49]. Radiation therapy modulates tumor phenotypes, enhances antigen presentation and tumor immunogenicity, increases production of cytokines and alters the tumor microenvironment, enabling destruction of the tumor by the immune system and making the tumor more susceptible to cytotoxic T lymphocyte-mediated cytotoxicity [49, 50]. The ‘immunogenic modulation’ capacity of radiation therapy promotes productive interaction between the tumor and the immune system, underlying its combination with immunotherapy in order to confer synergistic clinical benefits [50]. This theory has been partially verified in a phase III clinical trial, in which the anti-PD-L1 antibody durvalumab was administered following definitive chemoradiotherapy, showing a significant improvement in PFS compared with placebo in locally advanced NSCLC patients unselected for PD-L1. Stereotactic body radiation therapy (SBRT) is a noninvasive precision treatment that is effective and well-tolerated for medically inoperable patients with early stage NSCLC; in fact, SBRT is currently considered the standard of care in patients with unresectable early stage NSCLC [51]. Preclinical research and retrospective analysis have indicated that low dose radiation therapy (LDRT) combined with PD-1 inhibitors after SBRT conferred a potential synergistic anti-tumor effect [52]. Recently, SBRT in combination with sintilimab has been studied in clinical trials.

In the dose escalation phase of a phase I study (NCT03812549), the synergistic anti-tumor effect of LDRT combined with sintilimab following SBRT was explored in 12 stage IV NSCLC patients, with primary endpoints of safety and tolerability [53]. The combination plan showed no dose-limiting toxicities, with no grade 3 or higher AEs, while the ORR among 10 evaluable patients was 70% [53]. Interestingly, the combination therapy increased the number of T cell clones and TCR diversity, and decreased the PD-1 expression on CD8 + T cells and circulating tumor DNA (ctDNA) in the patients’ peripheral blood samples [53]. With an acceptable safety profile, sintilimab plus radiation therapy is undergoing several clinical trials in lung cancer patients [54]. Granulocyte–macrophage colony stimulating factor (GM-CSF) plays a pivotal role in the differentiation and maturation of dendritic cells, and can serve as a potent immune adjuvant or sensitizer [55]. In 2019, a multicenter, single-arm, phase II trial SWORD/NCT04106180 was initiated to assess the safety and efficacy of SBRT followed by sintilimab plus GM-CSF [56]. Recently, the safety run-in result of SWORD has been released; in the study, 20 metastatic NSCLC patients who failed to respond to first-line chemotherapy were enrolled, and treated with SBRT (8 Gy × 3) to one lesion, followed by sintilimab and GM-CSF [57]. No dose-limiting toxicities were observed and 18 patients experienced TRAE; no TRAE-related deaths were observed [57]. The trial is ongoing and continues to recruit participants. The clinical benefit conferred by this therapy remains to be verified.

#### Sintilimab in combination with anlotinib

Anlotinib is a multi-target drug that can block angiogenesis and proliferation of tumors by inhibiting vascular endothelial growth factor receptors (1/2/3) and other major tyrosine kinase receptors [58]. It has shown anti-tumor effect and clinical benefit in Chinese patients with advanced NSCLC. In the phase II ALTER 0302 study and phase III ALTER 0303 study, the primary endpoints PFS and OS were both significantly longer in patients receiving anlotinib than those receiving placebo (median PFS 4.8 versus 1.2 months, *P* < 0.0001, in ALTER 0302; median OS 9.6 versus 6.3 months, *P* = 0.002, in ALTER 0303) [59, 60]. The synergistic anti-tumor effect of anlotinib and sintilimab was studied in a single-center, open-label, phase Ib study conducted at Shanghai Chest Hospital (NCT03628521). In this study, 22 inoperable or metastatic NSCLC patients without EGFR/ALK/ROS1 mutations were given sintilimab plus anlotinib until disease progression or unacceptable toxicity [61]. The two primary endpoints were ORR and safety. The ORR was achieved in 72.7% (16/22) of the patients and all patients achieved tumor shrinkage and disease control. Median PFS was 15 months; neither PD-L1 nor TMB was related to treatment response, while PD-L1 + patients had longer median PFS compared to PD-L1- patients (not reached versus 14 months) [61]. TRAEs occurred in all treated patients; 54.5% of patients developed grade 3 or higher TRAEs, and one case of grade 5 immune-related pneumonitis was reported [61]. Based on the results, an open label, multi-center, randomized control study led by Shanghai Chest Hospital to compare the combination of sintilimab and anlotinib with standard platinum-based chemotherapy in treatment-naive advanced NSCLC patients was conducted (SUNRISE/NCT04124731) [44]. The results of this study are anticipated.

### Ongoing trials at our center

Currently, there are three ongoing clinical trials investigating sintilimab combination therapies in NSCLC at our center. ChiCTR1900025651 is a phase II trial investigating anlotinib combined with sintilimab in the second-line treatment of advanced NSCLC [62]. ChiCTR1900023074 is a single-arm phase II clinical study exploring sintilimab in advanced NSCLC with EGFR/HER2 gene exon 20 insertion mutations [63]. ChiCTR2000034888 is a phase Ib/II multi-cohort study investigating chidamide combined with sintilimab in advanced and refractory NSCLC [64]. These trials will provide opportunities to gain further understanding of the features of sintilimab in clinical practice.

## Pathologic response

Despite the progress made in immunotherapy, surgical treatment still remains the optimal choice for early-stage NSCLC patients [19, 32]. Meanwhile, platinum-based chemotherapy plans are the preferred systemic therapy regimens recommended by the NCCN in a neoadjuvant setting and offered an extra 5% survival benefit and a 10% absolute benefit on distant recurrence for resectable NSCLC patients [4, 19]. After most types of systemic treatment, including chemotherapy, traditional RECIST response criteria are used to assess tumor response [19]. However, imageological evaluation after neoadjuvant immunotherapy can sometimes be challenging and misleading; when patients with actual pathologic response were reported to have PD, the inconsistency could be attributed significantly to pseudo-progression [19, 65]. Alternatively, pathologic response evaluation after neoadjuvant therapy provides a window to assess the anti-tumor efficacy and possible clinical benefits achieved in patients at an early time point [66]. MPR is defined as 10% or less residual viable tumour after neoadjuvant chemotherapy, and pCR is defined as the absence of any remaining active tumor cells [67]. Both MPR and pCR are recommended as surrogate endpoints for lung cancer in the era of neoadjuvant immunotherapy [67].

It has been demonstrated that neoadjuvant immunotherapy can improve pathological response rates with acceptable toxicities [68]. On average, in patients receiving ICIs as neoadjuvant therapy, 45.6% (159/349) exhibited MPR, while 21.8% (76/349) achieved pCR [69]. For sintilimab in ChiCTR-OIC-17013726, the results are encouraging; the proportion of patients achieving MPR is 40.5%, that for pCR is 16.2%, and only two of the patients were delayed for surgery because of TRAEs [15]. The result was similar to that of a pilot study reported by Forde et al. (overall MPR rate of 45%, without surgery delay) [70]. The result is also in line with a superior 2-year DFS rate observed in patients who achieved MPR compared to those with non-MPR (86.7% versus 63.8%) at the 2-year follow-up, implicating that an improved prognosis was likely predicted by MPR [16].

Consistently, in the analyses of pathologic responses in surgical specimens obtained from 31 squamous NSCLC patients from ChiCTR-OIC-17013726, heterogeneous immune responses were identified among different residual viable tumor cell nests in the same tumor. In eight of nine MPR specimens, the residual viable tumor showed a 100% immune-activated phenotype; the remaining one MPR specimen exhibited an 80% immune-activated phenotype. In contrast, in specimens with partial pathologic response, a 60% immune-excluded/desert phenotype was observed, and an 80% immune-excluded/desert phenotype was found in specimens with no pathologic response. Meanwhile, three of six patients with pCR had lymph node metastasis [71]. These results indicate that assessment of pathologic responses in primary tumors could be a surrogate index for assessing the efficacy of neoadjuvant immunotherapy and that assessment of pathologic response in lymph nodes is also important.

## Predictive biomarkers

The proportion of patients that benefit from ICIs is limited. In the current age of personalized medicine, better characterization of patients with poor outcomes during immunotherapy remains an unmet need [72]. Under these circumstances, identification of effective predictive biomarkers of response may enable the production of better treatment decisions and provide an important reference for immune checkpoint inhibitor selection.

In the neoadjuvant setting (ChiCTR-OIC-17013726), predictive biomarkers have been explored. In this setting, MPR could serve as a surrogate efficacy biomarker, and superior 2-year DFS rates were observed in patients who achieved MPR (MPR versus non-MPR: 86.7% vs. 63.8%) [16].Patients with a higher TMB (≥ 10 mutations/Mb) and PD-L1 (TPS ≥ 50%) tend to have a higher 2-year DFS rate; patients with a TMB ≥ 10 mutations/Mb also had a significantly improved event free survival (HR = 0.125) [16]. Additionally, pre-immunotherapy treatment tumor samples of twenty-nine NSCLC patients from this group of patients were subjected to targeted DNA sequencing and PD-L1 immunochemistry staining, revealing that TPS was positively correlated with the degree of pathologic regression, TMB was not significantly correlated with pathologic regression, and that the copy number gain burden was significantly negative correlated with pathologic regression [73]. Moreover, a higher TPS (≥ 50%), a higher TMB (> 12 mutations/Mb), and a lower copy number gain burden was significantly correlated with MPR [15, 73]. For this group of patients, PET-CT was also performed at baseline and prior to surgery, and the metabolic information collected by FDG-PET was correlated with the surgical pathology, suggesting that ^18^F-FDG PET-CT can predict MPR to neoadjuvant sintilimab in resectable NSCLC [74].

To date, measurement of PD-L1 expressions by immunohistochemistry (IHC) is the only approved marker (category 1 recommendation) to select patients who are candidates for PD-1/PD-L1 inhibitors [19]. However, only a proportion of PD-L1 + patients respond to immunotherapy. Moreover, a high PD-L1 expression is not always correlated with tumor response [36, 61]. There are a number of possible reasons for this. The first is the differences of IHC assays across different studies (Agilent 22C3 pharmDx assay for ORIENT-12, 22C3 pharmDx for NCT03628521, CST13684S for ChiCTR-OIC-17013726, Dako clone 22C3 for NCT02937116, etc.), since the definition of a positive PD-L1 test result varies depending on which biomarker assay is used [75]. Secondly, most tissues used for PD-L1 detection are obtained from biopsy, while the actual correlation between the PD-L1 expression in tumor cells and immune cells between lung biopsies and corresponding resected tumors is poor [76]. Moreover, there is widespread intratumoral heterogeneity in NSCLC that lays the foundation for tumor evolution and drug resistance [77]. In addition, the dynamic nature of PD-L1 expressions leads to temporal variation, particularly after chemotherapy [78, 79]. As a consequence, more dependable indicators are warranted for prediction of tumor response.

TMB measures the number of somatic mutations in a tumor and is an emerging prognostic and predictive biomarker for immunotherapies such as anti-PD-1/PD-L1 therapy [80]. Studies have shown that a higher mutational burden in lung cancer is associated with improved objective response, durable clinical benefit, and better PFS [81]. However, inconsistencies are also observed between TMB and tumor response, where high TMB patients do not respond to immunotherapy [36, 61]. Possible explanations for the inconsistencies might be different cut-off values among the studies (4.25 mutations per Mb in NCT02937116 and 10 mutations per Mb in NCT03628521, for example), different test platforms (Illumina HiSeq X-Ten for NCT02937116 and FoundationOne CDx assay for NCT03628521, for example), and inadequate or insufficient tissue for testing [36, 61]. As a result, more effective biomarkers based on TMB are being explored.

Previously, a retrospectively study on blood TMB (bTMB) was conducted using pretreated NSCLC patients from the OAK and POPLAR studies; the study revealed that there was a positive correlation between bTMB and tissue TMB score and that bTMB could reliably identify patients who had significant clinical improvements in PFS due to anti-PD-L1 therapy as a second-line or higher treatment in NSCLC [82]. In addition, there are multiple soluble molecules that are potential biomarkers for NSCLC treated with immunotherapy [83]. More recently, a noninvasive multi-parameter assay used to predict NSCLC patients’ response to checkpoint inhibitors was developed; the study demonstrated that bTMB and ctDNA prior to treatment reciprocally correlated with clinical benefit, and their ratio was then integrated into a ‘normalized bTMB’. This ultimate multi-parameter biomarker comprised of normalized bTMB, CD8, ctDNA achieved excellent classification performance and could dependably predict patients who will achieve durable clinical benefit with a higher accuracy than any individual feature [84]. It is unknown how these biomarkers and models will perform in NSCLC, but this may be verified in future studies.

## Adverse effects

Despite the great success of immunotherapy, the increasing use of ICIs has resulted in incremental reports of adverse effects, making it a non-negligible issue [85]. Generally, adverse events are classified into five grades, which were defined according to the Common Terminology Criteria for Adverse Events from the National Cancer Institute, referring to mild, moderate, severe or life-threatening AEs, or death, in an ascending order [86]. The general safety of ICIs has been investigated in a previous meta-analysis incorporating 36 phase II/III trials, and the pooled incidence ranges from 54 to 76% for all AEs [87]. In addition, the incidences of fatal AEs for PD-1 inhibitors and PD-L1 inhibitors were 0.361% (33/9136) and 0.63% (12/3164), as revealed by a study that comprehensively evaluated the spectrum of fatal checkpoint inhibitor-related toxic effects [88]. For sintilimab, the incidence of TRAEs across the 12 studies ranges from 16.7 to 100%, and that of fatal TRAEs ranges from 1 to 6.25% (Fig. 2). The incidence of fatal TRAEs for sintilimab monotherapy ranges from 0 to 2.5% (1/40) (Fig. 2). The fatality rate caused by sintilimab seems to be higher than average; possible causes may be patient selection and potential differences in pharmacologic properties. The small sample size is also a possible cause of random errors. Nevertheless, this may be a reason that single-agent sintilimab has not been further tested in NSCLC.

It is noted that, when sintilimab was combined with platinum-based doublet chemotherapy, the incidence of all-grade TRAEs was 99.6% in ORIENT-11 and 100% in ORIENT-12, and the incidences of grade 3 or higher TRAEs were 61.7% and 86.6% [17, 18]. In a recent meta-analysis involving 161 studies and 17197 patients, when chemotherapy was combined with PD-1 or PD-L1 inhibitors, the overall incidence was 97.7% for all-grade TRAEs and 68.3% for grade 3 or higher TRAEs [89]. The safety profiles of the combination plans do not seem inferior to the average. This may be one of the important bases for their approvals by the NMPA [12, 14].

Interestingly, we noted that, in both studies, addition of sintilimab to chemotherapy decreased the incidence of TRAE-related fatality; the incidences were 2.3% (6/266) versus 6.9% (9/131) in ORIENT-11 and 4.5% (8/179) versus 6.7 (12/178) in ORIENT-12 [17, 18]. This was not clearly explained in either article. A recent study has illustrated that, while the addition of chemotherapy to immunotherapy increased the efficacy in NSCLC, it might also inhibit an overactive immune response and thereby reduce immune-related adverse events. In comparison with using a PD-L1 inhibitor alone, combination with chemotherapy for the first-line treatment of NSCLC could decrease the rates of most immune-related AEs [90]. This might be an explanation, since the baseline characteristics of patients in both groups in the two studies are similar; however, the exact underlying mechanisms are worthy of further exploration.

## Conclusions

The immense success achieved by monoclonal antibodies targeting T-cell inhibitory checkpoint receptors in stimulating anti-tumor immune responses in the treatment of cancer has dramatically changed cancer therapy [91]. Anti-PD-1/PD-L1 antibodies, either alone or with other systemic treatment, have significantly improved clinical benefit in patients with inoperable NSCLC [72]. However, only a certain proportion of patients benefit from ICIs, while others remain unresponsive or suffer from intolerable toxicities [83]. Therefore, novel ICIs or combination therapies may offer potential solutions to cancer patients. Moreover, despite the fact that a variety of ICIs contribute to improved survival for patients with advanced NSCLC, medical cost remains a major barrier to global access to these treatments. Although the cost-effectiveness of sintilimab and other PD-1inhibitors have not been compared yet, the successful development of economical PD-1 inhibitors like sintilimab will eventually lead to improved affordability of these agents for patients suffering from NSCLC. Under these circumstances, research on sintilimab may have added to another brick in the wall [92].

In general, sintilimab and its combinatorial plans were well tolerated in patients with NSCLC. Pathologic responses could act as surrogate endpoints for neoadjuvant immunotherapy and better predictive biomarkers are warranted. Single agent sintilimab was effective in a neoadjuvant setting in resectable NSCLC, but the effect became inconclusive when it was used in GGNs, which implicates the importance of appropriate timing in treatment. Combinations of sintilimab with GP or PP chemotherapy have conferred better anti-tumor efficacy and clinical benefits compared to GP or PP alone, while lowering treatment-related fatalities. Meanwhile, other combination plans, including sintilimab plus docetaxel, CIK, SBRT and LDRT, SBRT and GM-CSF, and anlotinib, showed promising anti-tumor effects. These results supported further investigation of sintilimab in future clinical trials.

## Data Availability

Not applicable.
